# Reference intervals for coagulation parameters in non-pregnant and pregnant women

**DOI:** 10.1038/s41598-022-05429-y

**Published:** 2022-01-27

**Authors:** Mengyu Fu, Junjie Liu, Jinfang Xing, Yanpeng Dai, Yanzi Ding, Kainan Dong, Xuewei Zhang, Enwu Yuan

**Affiliations:** 1 Department of Clinical Laboratory, the Third Affiliated Hospital of Zhengzhou University, No.7 Front Kangfu Street, Er’qi District, Zhengzhou, 450052 China; 2 Henan Human Sperm Bank, the Third Affiliated Hospital of Zhengzhou University, Zhengzhou, 450052 China

**Keywords:** Biomarkers, Medical research

## Abstract

Established reference intervals (RIs) of coagulation parameters generally based on the general population are not applicable to specific women. In order to accurately evaluate the coagulation status of non-pregnant women and pregnant women, specific RIs should be established. Our study recruited 465 non-pregnant women and 1972 pregnant women aged 20–45 years. Eight tests including antithrombin (AT), protein C (PC), free protein S (fPS), lupus anticoagulant (LA), D-dimer, fibrin/fibrinogen degradation products (FDP), coagulation factor VII (FVII), and factor VIII (FVIII) were performed on ACL TOP automated coagulation instrument. The RIs for these tests were established in non-pregnant and pregnant women at different gestational weeks. Compared to the non-pregnant group, the medians of AT and fPS were lower, while the medians of PC, LA normalized ratio, D-dimer, FDP, FVII, and FVIII were higher. During pregnancy, AT and fPS activity showed a decreasing trend, with the increase of gestational age. PC activity, LA normalized ratio, D-dimer concentrations, FDP concentrations, FVII, and FVIII activity presented an increasing trend, with the increase of gestational age. The non-pregnant women-specific RIs and the gestational age-specific RIs of AT, PC, fPS, LA normalized ratio, D-dimer, FDP, FVII, and FVIII needed to be established for accurate clinical diagnoses.

## Introduction

Pregnant women are in a hypercoagulable state, which is manifested not only by an increase of physiological pro-coagulation factors, a decrease of natural anticoagulant proteins, and changes of fibrinolytic system products^1–4^, but also may show the changes of pathological anticoagulant proteins, such as lupus anticoagulant (LA)^5^. Compared with non-pregnant women, the prethrombotic status of pregnant women increases the risk of developing thrombotic diseases, such as venous thromboembolism^6^. On the other hand, pregnancy complications and adverse pregnancy outcomes, such as preeclampsia and bleeding at delivery, have also been shown to be related to the procoagulant status^7,8^. Accurate evaluation and management of maternal coagulation status is a necessary prerequisite to ensure the health of pregnant women.

Appropriate RIs are the basis of clinical diagnosis, and more than 80% of clinical decisions depend on laboratory results^9^. Currently, most of the RIs for coagulation parameters used in clinical laboratories are derived from reagent manufacturers and are based on the general population. Whether these RIs apply to local non-pregnant and pregnant women has not been confirmed. Although there have been some papers establishing the RIs of coagulation factors in pregnant women, most studies focused on Caucasians, and studies on Chinese people are lacking. Some studies included only pregnant women of a certain period or did not include corresponding non-pregnant women as controls, and there was a lack of comparison between gestational weeks of pregnant women, and between pregnant women and non-pregnant women^2,10–12^. Secondly, in terms of methodology, some studies did not establish the RIs according to the standard method recommended by the Clinical and Laboratory Standards Institute (CLSI) C28-A3 document^13^, for example, the study sample size was less than 120 cases^2,11,14,15^. In aspect of experimental conditions, different instruments and reagents can affect the results of establishing the RIs for coagulation factors. Given the above factors, the RIs of coagulation parameters provided by the reagent manufacturer or in the literature are likely to fail to accurately diagnose coagulation diseases.

Therefore, clinical laboratories should establish RIs for coagulation parameters that are appropriate for the specific population in their region. The objective of our study was to establish RIs for antithrombin (AT), protein C (PC), free protein S (fPS), LA described as LA normalized ratio, D-dimer, fibrin/fibrinogen degradation products (FDP), coagulation factor VII (FVII) and factor VIII (FVIII) according to CLSI guidelines for non-pregnant and pregnant women at different gestational weeks in Henan Province, China.

## Results

**Figure 1:**
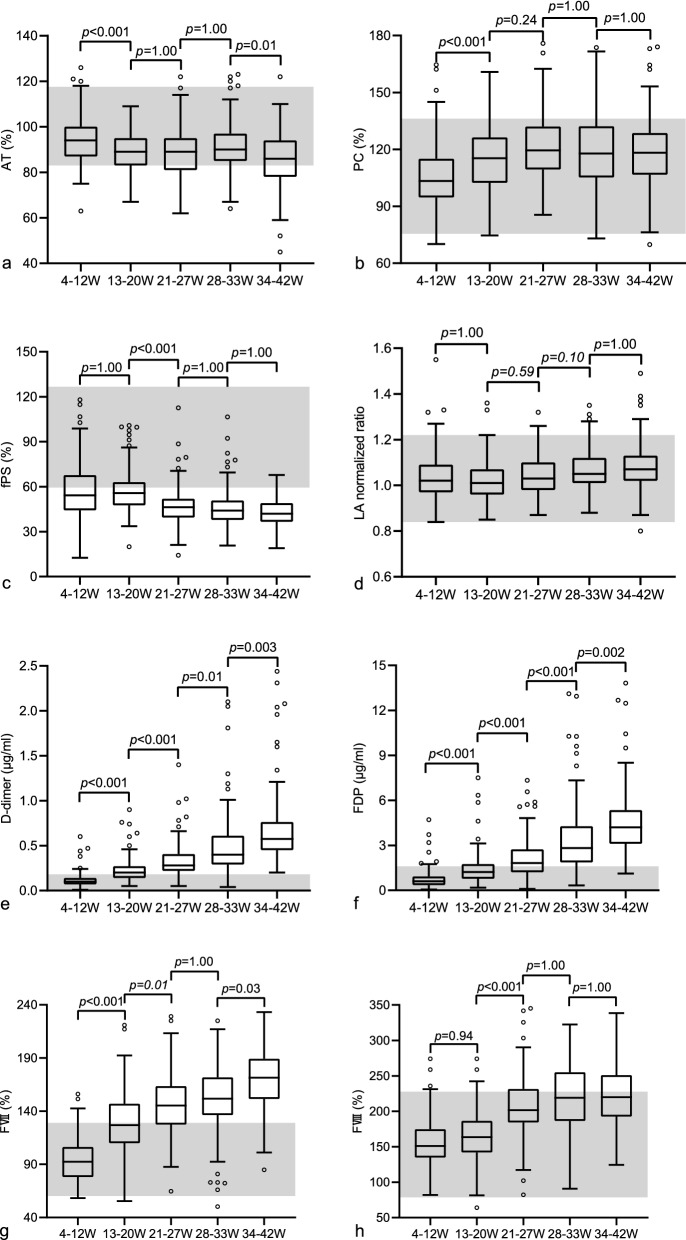
Box plot of gestational age-specific reference ranges for eight coagulation parameters. AT, antithrombin (**a**); PC, protein C (**b**); fPS, free protein S (**c**); LA normalized ratio, lupus anticoagulant normalized ratio (**d**); D-dimer (**e**); FDP, fibrin/fibrinogen degradation products (**f**); FVII, coagulation factor VII (**g**); FVIII, factor VIII (**h**); W, weeks; Each box plot includes the middle 50% of the data. The upper edge and the lower edge of the box indicate the 75th percentile and the 25th percentile, respectively. The bar in the middle of each box plot represents the median. The ‘whiskers’ extending from the box plot represent the range of values obtained excluding outliers. Circles outside the ends of the whiskers indicate outliers and extreme values. The shaded area represents the non-pregnant reference intervals in this study. *P *is the comparison between the two groups of pregnancy.

A total of 2437 women were recruited in the study, including 1972 pregnant women with no complications and 465 healthy non-pregnant women. The Kolmogorov–Smirnov test indicated that the distribution of the age, gestational age, AT, PC, fPS, LA normalized ratio, D-dimer, FDP, FVII, and FVIII were not normally distributed. The median age and gestational age of subjects in different groups are shown in Table 1. There was no significant difference between the median age at 4–12, 13–20, 21–27, 28–34, 35–42 weeks of gestation and the non-pregnant group (*P* > 0.05). There was also no difference between 4–12, 13–20, 21–27, 28–33, and 34–42 weeks of gestation. After analysis of the Dixon D/R ratio, there were two outliers presented in Table 2. The trends for AT, PC, fPS, LA normalized ratio, D-dimer, FDP, FVII, and FVIII were shown in Fig. 1. The median values, the RIs (2.5th and 97.5th percentiles) with 90% confidence intervals for non-pregnant women and each group of pregnant women were presented in Table 2. The number and proportion in each gestational group exceeding the upper or lower limit of the non-pregnant RI was shown in Table 3.

**Table 1:** Characteristics of non-pregnant and pregnant women.

Test (units)	*N*	Groups	Median age [IQR] (Y)	Median gestational age [IQR] (W)
AT (%); PC (%); fPS (%); LA normalized ratio	209	Non-pregnancy	30.00(6.00)	–
	237	4–12 W	30.00[5.00]	10.00[5.00]
	156	13–20 W	30.00[5.00]	16.00[4.00]
	168	21–27 W	29.00[4.00]	24.00[3.00]
	175	28-33 W	30.00[5.00]	31.00[2.00]
	163	34–42 W	30.00[4.00]	36.00[3.00]
D-dimer (μg/ml); FDP (μg/ml)	278	Non-pregnancy	30.00[5.00]	–
	297	4–12 W	30.00[5.00]	10.00[4.00]
	198	13–20 W	30.00[5.00]	16.00[4.00]
	168	21–27 W	29.00[4.00]	24.00[4.00]
	192	28-33 W	30.00[4.00]	31.00[3.00]
	206	34–42 W	30.00[4.25]	37.00[2.00]
FVII (%); FVIII (%)	125	Non-pregnancy	30.00[5.50]	–
	147	4–12 W	30.00[5.00]	10.00[2.00]
	132	13–20 W	31.00[7.00]	17.00[4.75]
	134	21–27 W	30.00[5.00]	24.00[3.00]
	126	28-33 W	30.00[6.00]	30.00[2.25]
	128	34–42 W	30.00[4.00]	37.00[2.00]

*AT* antithrombin, *PC* protein C, *fPS* free protein S, *LA normalized ratio* lupus anticoagulant normalized ratio, *FDP* fibrin/fibrinogen degradation products, *FVII* factor VII, *FVIII* factor VIII, *W* weeks, *Y* years, *IQR* interquartile range.

**Table 2:** Medians, RIs (the 2.5th and 97.5th percentiles) with 90% confidence intervals of non-pregnant and pregnant women.

Test (units)	Groups	*N*	Median	Lower limit (*CI*)	Upper limit (*CI*)
AT (%)	Non-pregnancy	209[0]	101.0	83.00(80.00–87.30)	117.50(114.00–120.00)
	4–12 W	237[0]	94.0	78.90(76.00–80.00)	116.00(111.25–120.00)
	13–20 W	156[0]	89.0	73.93(71.63–76.00)	105.08(102.23–106.23)
	21–27 W	168[0]	89.0	67.23(62.00–71.23)	109.78(108.00–116.97)
	28–33 W	175[1]	90.0	69.38(67.00–72.75)	117.63(107.88–121.25)
	34–42 W	143[0]	86.0	60.80(49.20–66.00)	109.40(107.00–114.80)
PC (%)	Non-pregnancy	209[0]	101.30	75.55(71.20–79.77)	136.20(132.35–140.00)
	4–12 W	237[0]	103.40	78.45(76.60–83.00)	136.16(133.94–151.20)
	13–20 W	156[0]	115.30	85.67(82.47–88.02)	154.96(148.67–158.67)
	21–27 W	168[0]	119.50	88.89(86.50–91.85)	159.76(150.00–168.93)
	28–33 W	175[0]	117.90	81.34(75.60–96.00)	161.60(154.66–168.42)
	34–42 W	143[0]	118.20	79.08(74.04–86.70)	163.12(148.30–173.46)
fPS (%)	Non-pregnancy	209[0]	88.80	59.48(52.00–64.30)	126.65(120.15–132.60)
	4–12 W	237[0]	54.30	26.09(24.70–31.62)	97.38(88.32–107.01)
	13–20 W	156[0]	55.75	34.90(32.58–38.97)	97.76(86.19–99.97)
	21–27 W	168[0]	46.35	28.60(21.10–30.55)	76.95(68.42–86.48)
	28–33 W	175[0]	44.10	24.74(23.20–27.80)	77.32(64.80–88.30)
	34–42 W	143[0]	42.00	25.94(21.58–29.00)	64.80(61.62–67.38)
LA normalized ratio	Non-pregnancy	209[0]	0.97	0.84(0.83–0.86)	1.22(1.15–1.25)
	4–12 W	237[0]	1.02	0.88(0.87–0.90)	1.21(1.17–1.32)
	13–20 W	156[0]	1.01	0.87(0.86–0.90)	1.21(1.19–1.33)
	21–27 W	168[1]	1.03	0.91(0.88–0.92)	1.21(1.20–1.26)
	28–33 W	175[0]	1.05	0.92(0.90–0.93)	1.28(1.25–1.31)
	34–42 W	143[0]	1.07	0.90(0.84–0.93)	1.36(1.25–1.43)
D-dimer (μg/ml)	Non-pregnancy	278[0]	0.08	0.01(0.01–0.02)	0.18(0.17–0.31)
	4–12 W	297[0]	0.10	0.02(0.02–0.03)	0.24(0.23–0.38)
	13–20 W	198[0]	0.20	0.07(0.06–0.09)	0.60(0.42–0.76)
	21–27 W	168[0]	0.28	0.09(0.08–0.14)	0.81(0.60–1.01)
	28–33 W	192[0]	0.40	0.16(0.08–0.19)	1.21(0.90–2.04)
	34–42 W	206[0]	0.58	0.29(0.22–0.31)	1.91(1.20–2.19)
FDP (μg/ml)	Non-pregnancy	278[0]	0.54	0.10(0.09–0.10)	1.60(1.55–1.97)
	4–12 W	297[0]	0.61	0.10(0.10–0.10)	1.75(1.70–2.54)
	13–20 W	198[0]	1.22	0.28(0.19–0.43)	3.45(2.84–6.34)
	21–27 W	168[0]	1.82	0.36(0.18–0.52)	5.70(4.68–6.57)
	28–33 W	192[0]	2.82	0.71(0.37–1.03)	9.73(7.06–12.83)
	34–42 W	206[0]	4.21	1.52(1.38–1.93)	9.32(7.97–12.48)
FVII (%)	Non-pregnancy	125[0]	83.80	60.30(51.43–62.11)	129.01(118.40–133.70)
	4–12 W	147[0]	92.40	61.07(58.72–63.94)	141.03(136.40–152.81)
	13–20 W	132[0]	126.90	70.64(59.24–80.00)	192.40(183.00–219.67)
	21–27 W	134[0]	145.20	92.40(73.33–102.20)	210.45(189.20–227.46)
	28–33 W	126[0]	151.75	72.34(52.98–93.89)	209.68(199.00–222.32)
	34–42 W	128[0]	171.50	110.16(88.45–120.31)	214.60(202.50–232.26)
FVIII (%)	Non-pregnancy	125[0]	135.80	78.62(74.36–84.10)	227.36(205.26–277.60)
	4–12 W	147[0]	151.00	94.36(90.26–98.70)	232.69(223.00–263.43)
	13–20 W	132[0]	163.60	98.95(69.72–110.90)	241.69(232.91–263.73)
	21–27 W	134[0]	201.60	118.33(89.73–127.44)	317.28(276.50–343.96)
	28–33 W	126[0]	218.95	132.43(97.00–152.40)	309.27(289.63–320.89)
	34–42 W	128[0]	219.95	155.37(128.71–158.53)	321.80(304.42–337.04)

*AT* antithrombin, *PC* protein C, *fPS* free protein S, *LA normalized ratio* lupus anticoagulant normalized ratio, *FDP* fibrin/fibrinogen degradation products, *FVII* factor VII, *FVIII* factor VIII, *W* weeks, *RIs* reference intervals; the number of subjects are listed with numbers of detected outliers (in square brackets).

**Table 3:** Number and proportion of each gestational group exceeding the upper or lower limit of the non-pregnant RIs.

Test	4–12 W, n/*N* (%)	13–20 W, n/*N* (%)	21–27 W, n/*N* (%)	28–33 W, n/*N* (%)	34–42 W, n/*N* (%)
AT < 83.00%	26/237(10.97)	36/156(23.08)	54/168(32.14)	30/174(17.24)	59/143(41.26)
PC > 136.20%	5/237(2.11)	21/156(13.46)	31/168(18.45)	36/175(20.57)	21/143(14.69)
fPS < 59.48%	143/237(60.34)	97/156(62.18)	153/168(91.07)	160/175(91.43)	132/143(92.31)
LA normalized ratio > 1.22	5/237(2.11)	2/156(1.28)	4/167(2.40)	12/175(6.86)	11/143(7.69)
D-dimer > 0.18 μg/ml	31/297(10.44)	108/198(54.55)	144/168(85.71)	185/192(96.35)	206/206(100)
FDP > 1.60 μg/ml	24/297(8.08)	61/198(30.81)	99/168(58.93)	157/192(81.77)	201/206(97.57)
FVII > 129.01%	11/147(7.48)	61/132(46.21)	99/134(73.88)	103/126(81.75)	119/128(92.97)
FVIII > 227.36%	5/147(3.40)	7/132(5.30)	40/134(29.85)	53/126(42.06)	48/128(37.50)

*AT* antithrombin, *PC* protein C, *fPS* free protein S, *LA normalized ratio* lupus anticoagulant normalized ratio, *FDP* fibrin/fibrinogen degradation products, *FVII* factor VII, *FVIII* factor VIII, *W* weeks, *RIs* reference intervals; n, number of gestational groups exceeding the upper or lower limit of the non-gestational RI, *N* number of pregnant groups.

During pregnancy, AT activity gradually declined during the first 20 weeks of gestation, and then remained unchanged, until a further slight decline occurred at 34–42 weeks (Fig. 1). Compared with the non-pregnant group, the median of AT activity was lower in all groups of pregnant women (*P* < 0.001), but remained within the non-pregnant RI. At 34–42 weeks of gestation, approximately 41.26% of pregnant women had AT activity below the lower limit of the non-pregnant RI (Table 3).

PC activity increased slightly from 13 to 20 weeks of gestation and remained at this level until birth (Fig. 1). Compared with the non-pregnant group, the median of PC activity was higher at 13–20, 21–27, 28–33, and 34–42 weeks of gestation (*P* < 0.001), but remained within the non-pregnant RI. In the third trimester, 17.92% of pregnant women had PC activity exceeding the upper limit of the non-pregnant RI (Table 3).

The activity of fPS decreased significantly as early as 4–12 weeks of gestation and reached its lowest value at 34–42 weeks (Fig. 1). Compared with the non-pregnant group, the median of fPS activity was lower in all pregnant groups (*P* < 0.001), and exceeded the lower limit of non-pregnant RI. At 4–12 weeks of gestation, 60.34% of the pregnant women had fPS activity lower than the non-pregnant RI. By the 34–42 gestation weeks, this proportion increased to 92.31% (Table 3).

Throughout the pregnancy, the median value of LA normalized ratio increased slightly (Fig. 1). Compared with the non-pregnant group, the median value of LA normalized ratio was higher (*P* < 0.001), but remained stable within the non-pregnant RI (Fig. 1). At 34–42 weeks of gestation, only 7.69% of pregnant women exceeded the upper limit of non-pregnant RI (Table 3).

D-dimer concentrations increased dramatically with the increasing gestational age (Fig. 1). Compared with the non-pregnant group, the median D-dimer was higher in all pregnant groups (*P* < 0.05). At 13–20 gestational weeks, the median value for D-dimer was higher than the upper limit of the non-pregnant RI. By 34–42 weeks of gestation, 100% pregnant women had D-dimer above the upper limit of the non-pregnant RI (Table 3).

FDP concentration increased significantly from 13 to 20 weeks, reaching a peak at 34–42 weeks (Fig. 1). Compared with non-pregnant group, the median of FDP was higher at 13–20, 21–27, 28–33, and 34–42 weeks of gestation (*P* < 0.001). At 21–27 weeks of gestation, the median FDP exceeded the upper limit of the non-pregnant RI, and 58.93% of pregnant women had FDP above the non-pregnancy upper limit. By 34–42 gestational weeks, this proportion increased to 97.57%. Moreover, the inter-quartile range of D-Dimer and FDP increased in the third trimester, and showed some maxima in Fig. 1.

FVII activity increased markedly from 13 to 20 weeks, reaching a maximum of 34–42 weeks (Fig. 1). Compared with the non-pregnant group, the median of FVII was higher at 13–20, 21–27, 28–33, and 34–42 weeks of gestation (*P* < 0.001). At 21–27 weeks of gestation, the median of FVII above the upper limit of non-pregnant RI. By 34–42 weeks of gestation, 92.97% of pregnant women had FVII activity above the upper limit of non-pregnant RI (Table 3).

FVIII activity increased gradually, reaching a peak at 28–34 weeks of gestation and then remained unchanged (Fig. 1). Compared with the non-pregnant group, the median FVIII was higher in all pregnant groups (*P* < 0.05), but remained within the non-pregnant RI. In the third trimester, 39.76% of pregnant women had FVIII exceeding the upper limit of the non-pregnancy RI (Table 3).

The results of trend test for coagulation indexes with gestational were shown in Table 4. With the increase of gestational age, AT and fPS activity showed a decreased trend (*P* = 0.022, *P* = 0.001), while PC activity, LA normalized ratio, D-dimer concentrations, FDP concentrations, FVII activity, and FVIII activity showed an increased trend (*P* < 0.001).

**Table 4:** The Chi-square trend test of coagulation indexes with gestational age.

Test (units)	*χ*^*2*^	*P*
AT (%)	5.223	0.022
PC (%)	39.000	< 0.001
fPS (%)	10.753	0.001
LA normalized ratio	25.124	< 0.001
D-dimer (μg/ml)	169.279	< 0.001
FDP (μg/ml)	209.087	< 0.001
FVII (%)	105.508	< 0.001
FVIII (%)	66.111	< 0.001

*AT* antithrombin, *PC* protein C, *fPS* free protein S, *LA normalized ratio* lupus anticoagulant normalized ratio, *FDP* fibrin/fibrinogen degradation products; *FVII* factor VII, *FVIII* factor VIII.

## Discussion

The balance of the coagulation system is a complex process affected by many factors. Coagulation parameters are not only affected by individual factors such as race, gender and pregnancy, but also by environmental factors, such as geography^16^, climate^17^, lifestyle, such as smoking^18^ and air pollution^19^. In the analysis of coagulation parameters, collection of specimens^20^, test methods, reagents, instruments, and operating specifications all affect the results. Although there have been some studies on the RIs of coagulation indexes, considering the influence of a variety of factors, each region should establish a local population-specific RIs.

Among the factors affecting coagulation indicators, gender is one of the most important factors. Some studies have confirmed that there are differences between male and female in coagulation indicators, including AT, PC, fPS, FVII and FVIII^21–23^. The study of Veen et al. confirmed that the use of routine RIs had a higher misdiagnosis rate than that of women-specific RIs^24^. Racial differences also affect coagulation indicators. The study of Ho et al. found that Chinese people have lower levels of PC and PS than Caucasians and other East Asians^25^. In addition, air pollution and smoking can cause a hypercoagulable state. Therefore, we screened the subjects strictly according to the inclusion and exclusion criteria and determined RIs for 8 coagulation factors for healthy non-pregnant women and uncomplicated pregnant women according to the CLSI C28-A3 document.

AT, PC and fPS are the crucial physiological anticoagulants in vivo. AT is the most important anticoagulant protein, accounting for 70% of anticoagulation. We found that AT activity decreased, showing a downward trend with the increase of gestational age, and reached the lowest level at 34–42 weeks of gestation. This was consistent with findings reported by Wang et al. and Cui et al., who also studied Chinese women^26,27^. However, some studies on Caucasians suggested that AT activity remained unchanged during pregnancy^2,10,28^. This difference may be due to ethnic and geographical differences. PC is another important inhibitor second only to AT. In our study, the PC activity increased slightly and showed an upward trend during pregnancy, but the median of PC remained within the nor-pregnant RI, which was consistent with some studies^10,29^. The slight increase in PC activity might be intended to neutralize thrombin generation at the maternal–fetal interface to maintain a normal pregnancy^10^. However, Wang et al.^26^ reported a gradual decrease in PC activity during pregnancy, whose subjects were people from Beijing, China. In their study, they used the same instruments and reagents as ours, but the results were completely different from our results. We speculated that this difference might be due to different lifestyles and different eating habits of subjects, selection bias of the study subjects, or factors related to laboratory operation, or other unknown reasons. PS plays an anticoagulant role through two mechanisms. One is that it acts as an important co-factor of PC to accelerate the inactivation of FVa and FVIIIa. On the other hand, PS can directly inhibit FXa and prothrombin^30^. As expected, the fPS activity decreased significantly from 4 to 12 weeks of gestation. It showed a downward trend with the increase of gestational age, which has been confirmed by some studies^2,11,26,31^. At 4–12, 13–20, 21–27, 28–33, and 34–42 weeks of gestation, the proportion of fPS activity exceeding the lower limit of non-pregnant RI was 60.34%, 62.18%, 91.07%, 91.43 and 92.31% respectively. If the RI of fPS activity for non-pregnant women, or the MRI based on the normal population, is used in the clinical setting, most pregnant women would be misdiagnosed as PS deficiency, which was consistent with the results reported by Szecsi et al. and Sekiyaet al.^2,15^. As a pathological anticoagulant protein, LA is one of the antiphospholipid antibodies. It is closely associated with thrombosis and pregnancy complications such as recurrent abortion and preeclampsia^8,32,33^. We found a slight increase in the median of LA normalized ratio during pregnancy, showed an upward trend with the increase of gestational age, in agreement with the results of Bokarewa et al.^5^. Although the median of LA normalized ratio remained within the non-pregnancy RI, we suspected that the slight increase could not be denied that it might further increase the hypercoagulable state of pregnant women.

As products of the fibrinolytic system, D-dimer and FDP are important screening indicators for the diagnosis of thrombotic diseases, such as venous thrombosis and disseminated intravascular coagulation. D-dimer is a marker of secondary fibrinolysis with a high negative predictive value. We confirmed that D-dimer increased dramatically with gestation, which is consistent with several studies^26,34–36^. At 13–20 weeks of gestation, the median value of D-dimer exceeded the upper limit of non-pregnancy. It showed an upward trend with gestational age. FDP is a marker of primary fibrinolysis, and the change of FDP during pregnancy was similar to that of D-dimer. At 21–27 gestation weeks, the median value of FDP exceeded the upper limit for non-pregnant women, similar to that reported by Wang et al.^27^. FDP concentration showed an upward trend with the increase of gestational age. Therefore, the non-pregnancy RIs of D-dimer and FDP have little clinical value for pregnant women and may contribute to misdiagnosis of thrombotic disease, which is consistent with the results of several studies^12,37,38^. In addition, we found that the inter-quartile range of D-dimer and FDP increased with increasing gestational age and showed some extreme values, especially in the third trimester. The diagnostic value of D-dimer and FDP was doubtful because the variation of D-dimer and FDP increased gradually with pregnancy, as also mentioned in studies of Szecsi et al. and Katrine et al.^2,39^.

The increase in FVII activity and FVIII activity during pregnancy is similar to the results of some studies, but the extent of their increase varies from study to study. FVII is a key factor of extrinsic pathway and its deficiency will lead to severe bleeding. Our study showed that FVII activity showed an upward trend with gestational age. At 21–27 gestation weeks, the median value of FVII activity exceeded the upper limit of the non-pregnant RI, in contrast to Szecsi et al. who reported FVII exceeded the upper limit of the non-pregnant RI at 29–34 gestation weeks^2^. This difference may be related to the instruments and reagents used in the laboratory, the race of the research subjects, and the geographical and climatic environment. FVIII is involved in endogenous coagulation, is a cofactor of factor IX, and is unstable in plasma. FVIII activity showed an upward trend with gestational age in this study. Some studies have reported a significant increase in FVIII as early as the first trimester^2,4^, whereas our results suggested that FVIII activity increased slightly during early pregnancy, and the median value of FVIII activity remained within the RI for non-pregnant women. Due to the instability of the eight factors, the differences in test methods and laboratory conditions can cause this difference.

Our study provided a relatively complete analysis of the changes of some coagulation factors in non-pregnant women and pregnant women at different gestational weeks and established the corresponding RIs. This method of establishing RIs through hospital data was proposed by Hoffmann^40^ and has been proved useful by other studies^9,41^. However, in order to prove the application value of the RIs for coagulation indicators in certain coagulation diseases in pregnant women, a large number of relevant case data are needed to verify. In addition, there are some limitations to our study, the most important of which is the lack of postpartum data. Secondly, the study population is only women in Henan province, which lacks sample diversity. Therefore, a multicenter study with a large sample size is required.

In conclusion, considering the influence of gender on coagulation, the non-pregnant women-specific RIs for coagulation indicators should be established. During pregnancy, AT activity, fPS activity decreased, PC activity, LA normalized ratio slightly increased, and D-dimer, FDP, FVII activity, and FVIII activity were significantly higher, confirming that pregnant women are in a hypercoagulation state. Considering the influence of multiple factors, and in order to accurate evaluation of pregnant women’s coagulation status, the gestational age-specific RIs of AT, PC, fPS, LA normalized ratio, D-dimer, FDP, FVII, and FVIII should be established.

## Materials and methods

### Subjects

**Figure 2:**
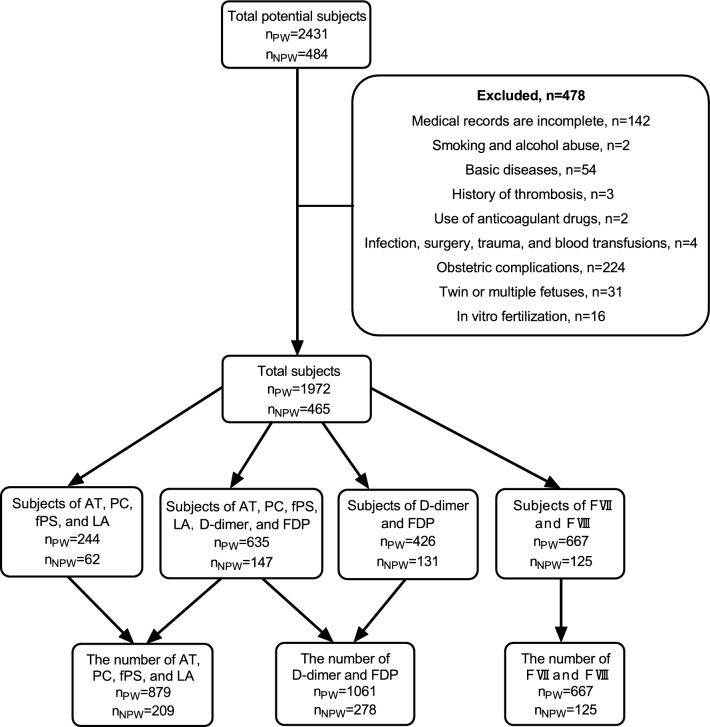
Flowchart of subjects included and excluded in the study. *AT* antithrombin, *PC* protein C, *fPS* free protein S, *LA normalized ratio* lupus anticoagulant normalized ratio, *D-dimer*,  *FDP* fibrin/fibrinogen degradation products, *FVII* coagulation factor VII, *FVIII* factor VIII; *N*_*PW*_ the number of pregnant women; n_NPW_ the number of non-pregnant women.

A total of 2431 pregnant women aged 20–45 years, including clinics and visits, were enrolled in the outpatient and inpatient Department of Obstetrics and Gynecology of the Third Affiliated Hospital of Zhengzhou University, from November 2019 to February 2021. All pregnant women of Han Chinese ethnicity are from Henan province, China. Aiming to select apparently healthy adult individuals, exclusion criteria were as follows: (1) medical records are incomplete; (2) smoking and alcohol abuse; (3) basic diseases (including heart, liver, kidney disease), hematologic system disease, thyroid disease, diabetes mellitus, and autoimmune disease; (4) personal or family history of thrombosis; (5) use of anticoagulant drugs, such as aspirin, heparin, etc.; (6) infection, surgery or trauma within 30 days, and a 6-month history of blood transfusions; (7) obstetric complications, such as pregnancy-induced hypertension syndrome, gestational diabetes mellitus, placenta abnormalities, etc.; (8) the twin or multiple fetuses; (9) in vitro fertilization-embryo transfer technology. All healthy pregnant women were assigned to 5 groups: 4–12, 13–20, 21–27, 28–33, and 34–42 weeks of gestation. This study also recruited 484 non-pregnant women aged 20–45 years, who underwent pre-pregnancy physical examinations from the outpatient department of obstetrics and gynecology, as the non-pregnant group. The exclusion criteria for non-pregnant women were the same as those for pregnant women in articles (1), (2), (3), (4), (5) and (6). The screening process of subjects was shown in Fig. 2. The methods were carried out in this study according to the relevant guidelines and regulations. All donor candidates were given an anonymous code number. Identifying information was known only to the staff of the clinical laboratory of the Third Affiliated Hospital of Zhengzhou University. This study was approved by the ethics committee of the Third Affiliated Hospital of Zhengzhou University. Written informed consent was obtained from all participants before conducting the study.

### Laboratory analysis

Blood samples were collected into vacuum tubes (Shandong Aosaite Medical Devices, Chengwu, Shandong) containing 1:9 volume of 0.109 mol/L trisodium citrate, and then centrifuged at 4000×*g* for 10 min at room temperature of 25 °C. Routine assays including the activity of AT, the activity of PC, the activity of fPS, LA normalized ratio tested using dilute Russell’s viper venom time (dRVVT) (LA normalized ratio = dRVVT screen ratio/dRVVT confirm ratio), D-dimer, and FDP. The resultant platelet-poor plasma of coagulation FVII and FVIII were stored at − 80 °C, until analysis. All tests were performed on the ACL TOP automated coagulation instrument (Instrument Laboratory, Bedford, USA), and the characteristics of the performed tests include manufacturer’s reference intervals (MRIs), test method, reagents information (Instrumentation Laboratory Co., USA), and stability of reagents were shown in Table 5. All coagulation tests were completed within 2 h. If the specimen had already been clotted, it would be excluded from the calculations.

**Table 5:** Characteristics of the performed tests.

Test (units)	MRI	Method	Reagent	Batch numbers	Stability (h)
AT (%)	83–128	Chromogenic assay	HemosIL Liquid Antithrombin kit	Cat.No.0020300400 Cat.No.0020030100	48
PC (%)	70–140	Chromogenic assay	HemosIL Protein C kit	Cat.No.0020300500	120
fPS (%)	63.5–149.0	Clotting time method (by turbidimetry)	HemosIL Protein S kit	Cat.No.0020302000	8
LA normalized ratio	< 1.2	Clotting time method (by turbidimetry)	HemosILdRVVT Screen and dRVVT Confirm kit	Cat.No.0020301500 Cat.No.0020301600	72
D-dimer (μg/ml)	< 0.24	Latex enhanced immunoassay	HemosIL D-Dimer HS kit	Cat.No.0020007700	96
FDP (μg/ml)	< 2.01	Latex enhanced immunoassay	HemosIL FDP kit	Cat.No.0020009900	72
FVII (%)	50–129	Clotting time method (by turbidimetry)	HemosIL Factor VII Deficient Plasma	Cat.No.0020011700	24
FVIII (%)	50–150	Clotting time method (by turbidimetry)	HemosIL Factor VIII Deficient Plasma	Cat.No.0020011800	4

*AT* antithrombin, *PC* protein C, *fPS* free protein S, *LA normalized ratio* lupus anticoagulant normalized ratio, *FDP* fibrin/fibrinogen degradation products, *FVII* factor VII, *FVIII* factor VIII, *MRI* manufacturer's reference interval, the test method and the batch number of each test are indicated. Stability time is presented when opened or reconstitution reagents stored at 2–8 °C.

### Statistical analysis

Statistical analysis was performed using the Statistical Package of Social Sciences, Version 21.0 statistical software. Box plots were used to identify the possible outliers. The outliers were excluded from each group when the D/R ratio was over 1/3^13^, where D is the absolute difference between an extreme value and the next largest value, and R is the range of all observations. The Kolmogorov–Smirnov test was used for normality tests. According to CLSI's recommendation^13^, when the sample size > 120, there is no need to assumption about data distribution. Therefore, RIs (2.5th and 97.5th percentile with 90% confidence intervals) were established for each coagulation parameter using a nonparametric method. Differences in the media were assessed by the Kruskal–Wallis test and the Mann–Whitney U test. The Chi-square trend test was used to analyze the trend of coagulation indexes with gestational age. The results were interpreted as statistically significant if the 2-tailed *P* < 0.05.

## Data Availability

The datasets used during the current study are available from the corresponding author on a reasonable request.
